# Supplementary material for: Predicting visual function by interpreting a neuronal wiring diagram
Source: Nature. 2024 Oct 2;634(8032):113–23. doi: 10.1038/s41586-024-07953-5 (PMC11446822; doi:10.1038/s41586-024-07953-5)
Supplement: Supplementary file 6 — CRF and ERF predictions for individual TmY4 and TmY9 cells. Analogous to Supplementary Data 3, but for TmY target types. Shown are the top four monosynaptic pathways, the strongest pathway passing through each of the top ten intermediary types (ranking from Extended Data Fig. 7), and the trisynaptic pathway Tm1–TmY–Dm3–TmY (see the section entitled Prediction of spatial normalization). [file 41586_2024_7953_MOESM6_ESM.zip › DataS4/TmY9qΓèÑ/720575940640290109.pdf]

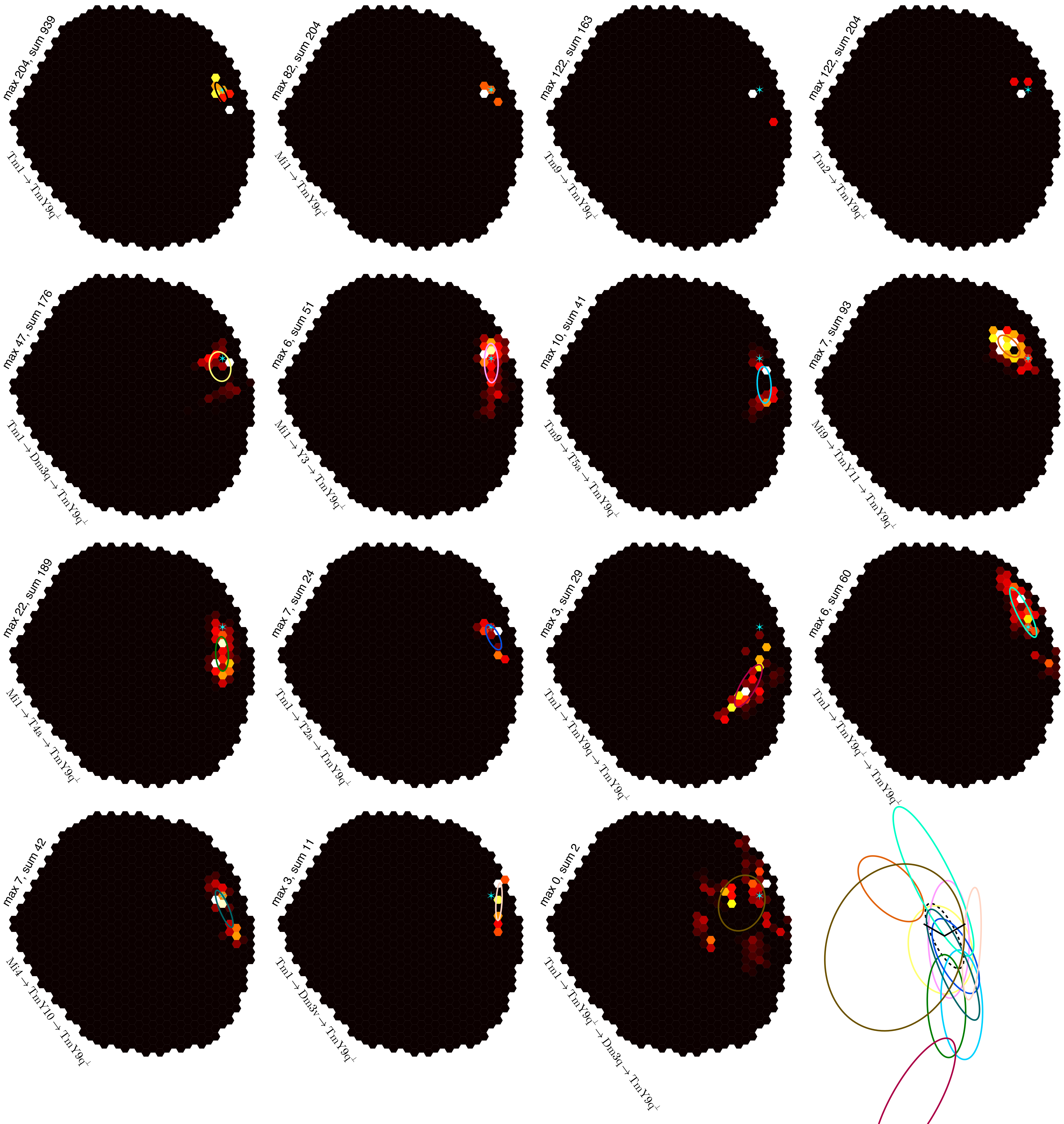
max 204, sum 939
Tm1 → TmY9q⊥
max 82, sum 204
Mi1 → TmY9q⊥
max 122, sum 163
Tm9 → TmY9q⊥
max 122, sum 204
Tm2 → TmY9q⊥
max 47, sum 176
Tm1 → Dm3q → TmY9q⊥
max 6, sum 51
Mi1 → Y3 → TmY9q⊥
max 10, sum 41
Tm9 → T5a → TmY9q⊥
max 7, sum 93
Mi9 → TmY11 → TmY9q⊥
max 22, sum 189
Mi1 → T4a → TmY9q⊥
max 7, sum 24
Tm1 → T2a → TmY9q⊥
max 3, sum 29
Tm1 → TmY9q → TmY9q⊥
max 6, sum 60
Tm1 → TmY9q⊥ → TmY9q⊥
max 7, sum 42
Mi4 → TmY10 → TmY9q⊥
max 3, sum 11
Tm1 → Dm3v → TmY9q⊥
max 0, sum 2
Tm1 → TmY9q⊥ → Dm3q → TmY9q⊥
